# A compartmentalized approach to the assembly of physical maps

**DOI:** 10.1186/1471-2105-10-217

**Published:** 2009-07-15

**Authors:** Serdar Bozdag, Timothy J Close, Stefano Lonardi

**Affiliations:** 1 National Cancer Institute, National Institutes of Health, Bethesda, MD 20892, USA; 2 Department of Botany and Plant Sciences, University of California, Riverside, CA 92521, USA; 3 Department of Computer Science and Engineering, University of California, Riverside, CA 92521, USA

## Abstract

**Background:**

Physical maps have been historically one of the cornerstones of genome sequencing and map-based cloning strategies. They also support marker assisted breeding and EST mapping. The problem of building a high quality physical map is computationally challenging due to unavoidable noise in the input fingerprint data.

**Results:**

We propose a novel compartmentalized method for the assembly of high quality physical maps from fingerprinted clones. The knowledge of genetic markers enables us to group clones into clusters so that clones in the same cluster are more likely to overlap. For each cluster of clones, a local physical map is first constructed using FingerPrinted Contigs (FPC). Then, all the individual maps are carefully merged into the final physical map. Experimental results on the genomes of rice and barley demonstrate that the compartmentalized assembly produces significantly more accurate maps, and that it can detect and isolate clones that would induce "chimeric" contigs if used in the final assembly.

**Conclusion:**

The software is available for download at

## Background

A physical map is a linear ordering of a set of overlapping clones in a genomic library. Physical maps are obtained from processing the signatures or *fingerprints *of all the clones in a library. Fingerprints can be generated by digesting clones with one or more restriction enzymes, or by hybridizing them to a carefully designed set of DNA probes. The computational problem is to build an overlap map of the clones that is consisted with the fingerprint data [1].

Physical maps have been historically one of the cornerstones of genome sequencing projects. For instance, in *clone-by-clone sequencing*, first a physical map is constructed; then, a minimum-cardinality set of overlapping clones that spans the genomic region represented by the physical map, called *minimal tiling path *(MTP), is selected. Finally, the clones in the MTP are sequenced one by one [2]. The clone-by-clone sequencing method has been used to sequence several genomes including *C. elegans *[3], *A. thaliana *[4], *H. sapiens *[5], and *O. sativa *[6]. In several recent whole-genome shotgun sequencing projects, physical maps have also been employed to validate and improve the quality of sequence assembly [7]. This validation step has been used, for example, in the assembly of *M. musculus *[8], *G. gallus *[9], and *O. anatinus *[10].

The rapid market penetration of next-generation sequencing instruments (Roche/454, Illumina, and ABI SOLiD) is expected to bring physical mapping back to the center stage of genomics. Next-gen sequencing technologies produce massive amounts of short reads (about 200–300 bases for 454, 35 bases for Illumina and SOLiD) [11] and therefore the *de novo *assembly of the whole eukaryotic genomes is extremely challenging [12]. Arguably, the only realistic approach at this time is clone-by-clone sequencing, where each clone in the MTP is sequenced using next-gen technology, and the assembly is carried out separately clone by clone (see [12-14] and references therein).

In addition to their prominence in sequencing projects, physical maps can also provide a robust infrastructure required by many applications in genomics such as marker assisted breeding [15], map-based cloning of a set of genes of interest [16]. and EST mapping [17].

Physical maps can be built from data obtained by restriction digestion or hybridization experiments [1]. In the former case, overlaps between clones are determined by a statistical method, then clones are arranged in an order that is consistent with the restriction fingerprint data [18]. In the latter case, clone-probe associations (i.e., which clones hybridize to which probe) are used to find an arrangement of the probes such that clones can be ordered consistently [19]. In practice, however, hybridization experiments rarely use single probes. Due to the time and cost involved, hybridizations between probes and clones are typically carried out for a pool of probes (see, e.g., [20]). In this work, we assume that only clone-pool associations (hereafter called *hybridization fingerprints*) are available.

Nowadays almost all physical mapping projects that are based on restriction fingerprint data rely on a tool called FingerPrinted Contigs (FPC) [18]. FPC implements an algorithm called *consensus band *(CB) that constructs a physical map using a combination of greedy and heuristic approaches. At the core of the CB algorithm, clones are assigned to contigs based on a coincidence score, called *Sulston *score, which measures the probability that two clones share a given number of restriction fragments (*bands*) according to a binomial probability distribution [21]. Two bands are considered *shared *if their sizes are within a given *tolerance *value. Two clones are declared *overlapping *if their Sulston score is below a given *cutoff *threshold. For each contig, FPC builds a CB map, which is a coordinate system to which clones are aligned.

FPC does not attempt to resolve all the conflicts arising in the assembly of the physical map, but instead provides interactive features for manual editing. Although manual editing appears to be an unavoidable final step in any physical mapping project, this process is tedious, very time-consuming and requires a significant expertise. Obviously, the better the initial quality of the physical map produced by the algorithm, the smaller is amount of manual work involved.

### Our contribution

With the objective of producing more accurate physical maps, here we propose a novel algorithmic pipeline that is capable of integrating both restriction and hybridization fingerprints. We note that the availability of both types of data is common in large-scale genomic projects. In this paper, the hybridization fingerprint data is obtained by hybridizing pools of short oligonucleotide probes to a Bacterial Artificial Chromosome (BAC) clone library [20].

**Figure 1:**
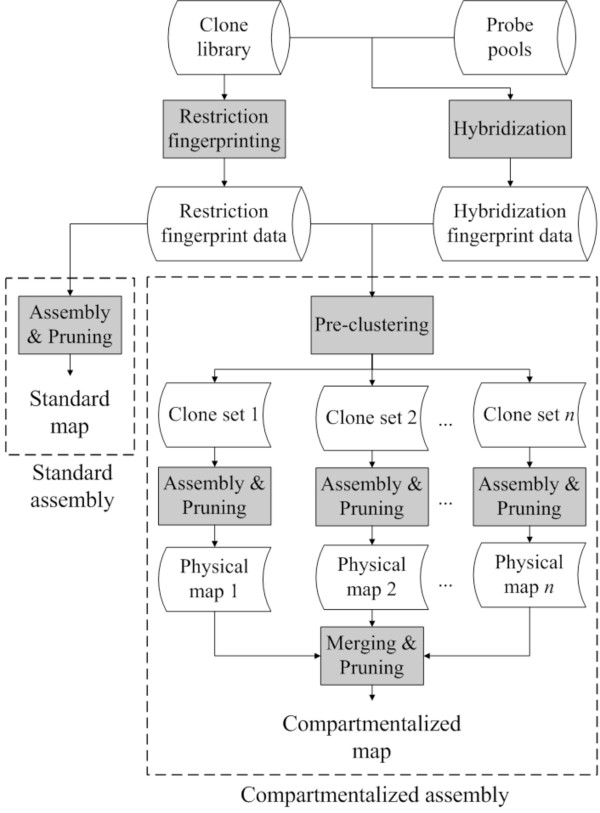
**A schematic overview of standard and compartmentalized contig assembly**.

While the typical use of FPC is to process the entire set of fingerprinted clones at once (approach hereafter called *standard *assembly), a *compartmentalized *assembly is feasible when hybridization fingerprint data is available. In the compartmentalized approach, we first pre-cluster clones based on their hybridization fingerprints. The purpose of the pre-clustering step is to group together clones that are more likely to be truly overlapping thus mitigating the effect of the noise in restriction fingerprint data, which is responsible for producing mis-assembled contigs [22]. Then, a "local" physical map of the clones in each cluster is built based on the restriction fingerprint data. Finally, all the local maps are merged into the final global physical map, which is pruned afterwards to remove redundancies and/or inconsistencies. An illustration of the compartmentalized and standard assembly is shown in Figure 1.

Given the trust of the scientific community in FPC, we decided not to "reinvent the wheel" but include some of its modules in our algorithmic pipeline as subroutines. In order to integrate FPC in our workflow, some minor changes to its source code had to be carried out. We use FPC to compute the local assemblies on each cluster of clones; we also employ jointly FPC and a novel algorithm described later to merge contigs iteratively. FPC's merge process is based on shared bands between contigs, whereas our algorithm is based on shared clones between contigs. In general, the strategy behind the design of our assembler is "be conservative first". For example, in the beginning of the assembly process, we merge contigs only when there is strong evidence that they overlap while later we allow riskier moves.

We tested our assembler extensively on several data sets. In the experimental section, we report results on the assembly of the physical map of two important crop plants, namely rice and barley. Real fingerprinting data is available for both plants, but real hybridization data is available only for barley, while rice hybridization data was simulated *in silico*. We constructed physical maps using standard and compartmentalized assembly for both plants, and evaluated different pre-clustering strategies for our assembler. We compared the accuracy of the maps produced by the two methods using a variety of metrics. We also compared the rice maps to the manually edited physical map of rice. Our evaluations show that the compartmentalized assembly produces significantly more accurate maps than maps produced by the standard assembly. In addition, our method is capable of detecting and isolating clones that would induce chimeric contigs if used by the standard assembly.

## Results and discussion

### Algorithm

#### Pre-clustering of clones

During the pre-clustering phase, clones that are more likely to be overlapping are assigned to the same cluster. We pre-cluster clones according to hybridization and/or restriction fingerprint data. Below are four pre-clustering approaches we implemented and analyzed.

#### Pre-clustering based on hybridization fingerprint data (HYB)

In HYB, overlapping clones are detected via an overlap score based on hybridization fingerprint data. Consider two clones *c*_*i *_and *c*_*j *_that hybridize to at least one of the probes in probe pool *p*. In this case, we say that *p *is a *positive pool *for *c*_*i*_, *c*_*j *_and that there is a *positive concordance *between the two clones. If both clones hybridize to none of the probes in probe pool *q *then we say that *q *is a *negative pool *for *c*_*i*_, *c*_*j *_and there is a *negative concordance *between the two clones. If one clone hybridizes to a probe pool but the other does not, then we say there is *discordance *between the two clones.

Our overlap score is based on two observations. First, observe that positive and negative concordance weigh in favor of clone overlaps, whereas discordance weighs against it. Second, observe that the strength of the positive concordance between two clones should be inversely proportional on the number probes in the pool. In fact, the probability that the two clones hybridize to the same probe (and therefore overlap) increases as the size of the probe pool decreases. Vice versa, the strength of the negative concordance between two clones should be directly proportional on the number probes in the pool. This is because as the negative pool size increases, the size of the region that the clones might occupy gets smaller and smaller (i.e., the probability that they reside in the same genomic region increases).

Based on these observations, we propose the following *positive-negative concordance (PNC) *score, which is defined between two clones *c*_*i *_and *c*_*j*_.



where *P*_*I *_and *P*_*U *_are the intersection and union of positive pools for the pair of clones *c*_*i*_, *c*_*j*_; *N*_*I *_and *N*_*U *_are the intersection and union of the negative pools for the pair of clones *c*_*i*_, *c*_*j*_; and |*p*| denotes the size of pool *p*. The PNC score ranges between zero and two, inclusively.

Any clustering algorithm can be used in combination with the PNC score. For simplicity, we used the single-linkage hierarchical clustering [23]. Consequently, disjoint (hard) clusters were generated (i.e., one clone can belong to exactly one cluster).

We performed extensive comparative evaluations of the proposed PNC score with other popular overlap scores, such as Sulston score [21], algebraically corrected Sulston score [24], Mott score [25], *weighted shared bands *score, and *positive concordance *score. The latter two scores are defined as follows. The *weighted shared bands *score of two clones is defined as the total weights of their shared bands. The *weight *of a band *b *is the fraction between total number of bands of all clones and frequency of bands of size between [*b *- *t*/2, *b *+ *t*/2], where *t *is the tolerance value. The weight of a band is inversely proportional to its frequency. The *positive concordance *score of two clones is defined as the fraction between the intersection and union of their positive pools (i.e., the first term of the PNC score).

**Figure 2:**
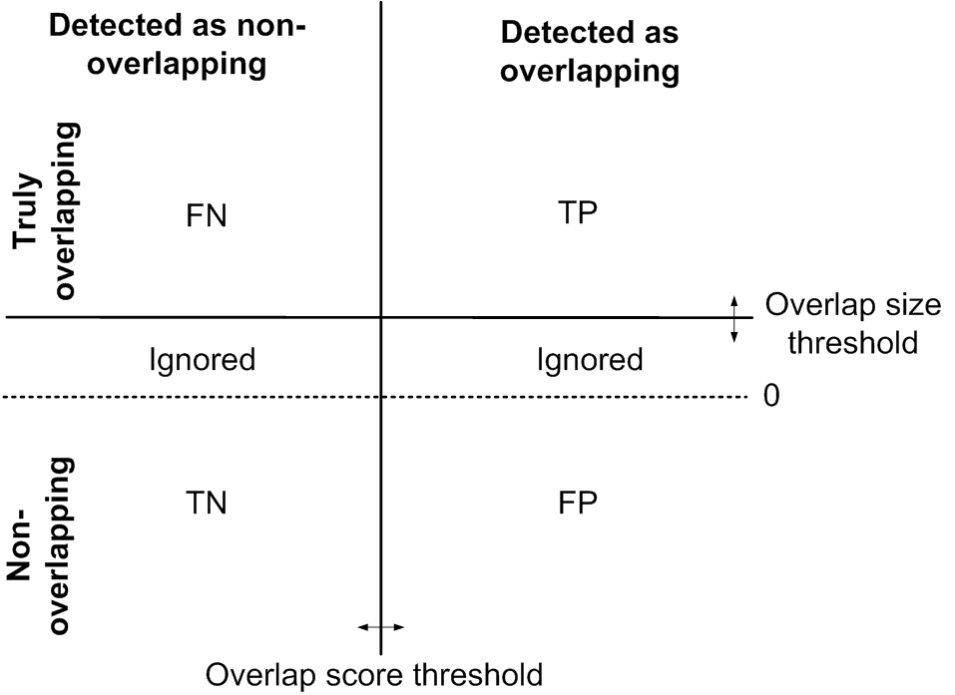
**ROC curve illustration**. An illustration of how ROC curves are generated. Each point in this coordinate system shows overlap score and size of a clone pair, and is classified as *TP*, *FN*, *FP*, *TN*, and *ignored *according to two given parameters, overlap score threshold and the overlap score type. Clone pairs whose overlap size is greater than zero, but less than the overlap score threshold are ignored.

**Figure 3:**
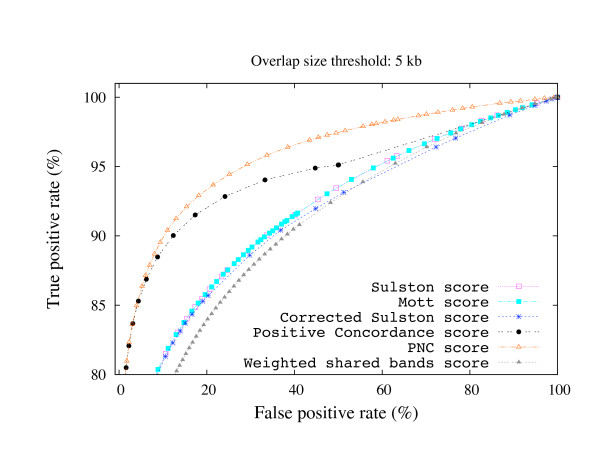
**Overlap score ROC curves (5 kb threshold)**. The ROC curves of several overlap scores for overlap size thresholds of 5 kb.

**Figure 4:**
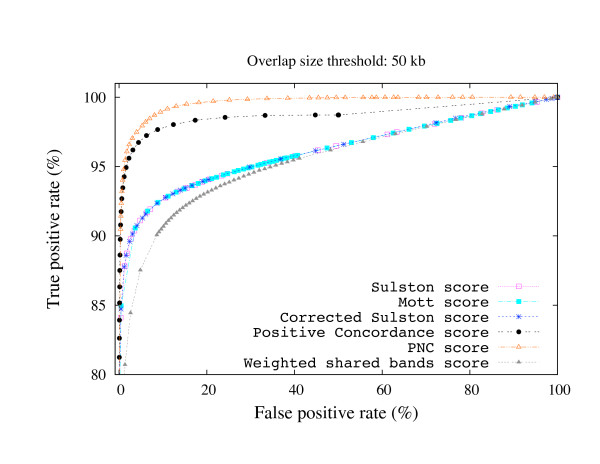
**Overlap score ROC curves (50 kb threshold)**. The ROC curves of several overlap scores for overlap size thresholds of 50 kb.

In order to assess the quality of the overlap scores, we collected all pairs of rice clones whose positions in the rice genome are known. For each pair we computed the overlap scores mentioned above and the overlap size. Then we computed the distribution of *true positive rate *(TPR) and *false positive rate *(FPR) for various overlap score and overlap size thresholds. The TPR and FPR can be computed as following.



where TP, FP, TN, and FN are true positive, false positive, true negative, and false negative overlaps as defined in Figure 2. Clone pairs whose overlap size is lower than the overlap size threshold, but greater than zero are ignored in this computation in order not to penalize/favor for missing/detecting "small" overlaps. Then, we fixed the overlap size threshold, and we computed the receiver operating characteristic (ROC) curve to plot the distribution of TPR and FPR as a function of the overlap score thresholds. The ROC curves for each overlap score are shown in Figure 3 and Figure 4 for overlap size thresholds of 5 kb and 50 kb, respectively. Observe that the PNC score outperforms the other scores in both cases. The improvement over the other scores is much higher with lower overlap size threshold values (i.e., it is more successful in detecting small overlaps than other overlap scores).

#### Soft pre-clustering based on hybridization fingerprint data (sHYB)

When probes are sparsely distributed in the genome, most clones have many more negative pools than positive pools. This generates negative concordances between many clone pairs whether they overlap or not. In addition, since the probability that a probe occurs in the overlap between two clones is very small, we expect only very few clone pairs to show positive concordance. Consequently, the PNC score will be close to one for most of the clone pairs whether they overlap or not.

In this case, a *soft clustering *might be more appropriate. In soft clustering, clones are assigned to multiple clusters when there is not sufficient evidence for a unique assignment. Initially, an empty cluster is generated for each pool. Then, clones are assigned to the clusters that correspond to their positive pools, i.e., clones that belong to the same pool are assigned to the same cluster. Finally, the clustering is finalized based on the restriction fingerprint data. When sHYB is used, redundant clones and/or redundant contigs can be present in the merged physical map. Additional steps are performed downstream to eliminate this redundancy (see Phase B in Physical Map Construction section).

#### Pre-clustering based on restriction fingerprint data (RESTR)

When no hybridization fingerprint data are available, one could consider pre-clustering clones based on the restriction fingerprint data. Note that the similarity between clones' fingerprints will be evaluated again during the actual contig assembly, so it is not obvious that pre-clustering using only restriction fingerprint data would bring any improvement in accuracy. For this choice of pre-clustering, we used the single-linkage hierarchical clustering to generate disjoint clusters by assigning clones with similar restricting fingerprint into the same cluster.

#### Random pre-clustering (RAND)

**Figure 5:**
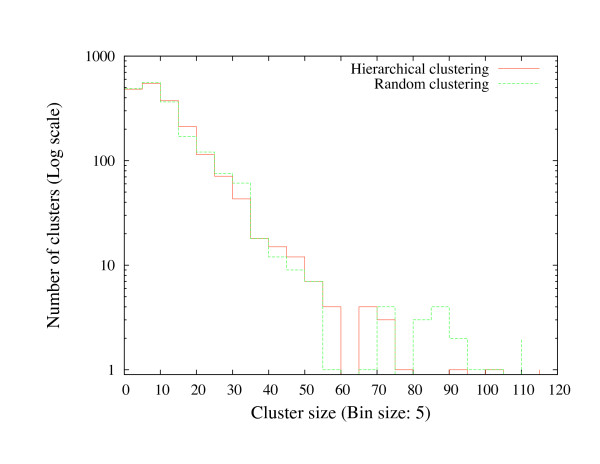
**Comparison of random clustering to hierarchical clustering**. The histogram of sizes of the rice clone clusters generated by single-linkage hierarchical clustering on the PNC score and random clustering.

We expect that a random pre-clustering would bring no benefits in the final assembly, so we can use RAND as a baseline on which to assess the efficacy of the other pre-clustering strategies on the compartmentalized method. The distribution of sizes of random clusters is based on the distribution of sizes for clusters generated by HYB method. As shown in Figure 5, the histograms of random and non-random cluster sizes are very similar.

In random clustering, first, the size of a cluster is determined randomly based on the size distribution of a cluster generated by HYB method and then clones that are not previously assigned to a cluster are assigned to this cluster randomly until the cluster size is reached. This step is repeated until all clones are assigned to some cluster.

#### Physical map construction

The first step in the compartmentalized method is to run FPC independently on each *clone set *(i.e., clusters) obtained in the pre-clustering phase. Observe that clone sets are not necessarily disjoint. Since FPC does not offer all of its functionalities in batch mode, we instrumented it so to enable batch mode processing of functions such as END-MERGER, DQER, and REBUILD-CONTIGS. We also added a user remark to identify questionable clones (Q-clones). A clone is called Q-clone if more than 50% of its bands do not align to the CB map (e.g. chimeric clones, which are formed by merging two or more non-overlapping clones during cloning) [26]. No other modification to the internal code of FPC was performed. FPC's key parameters such as cutoff, tolerance, and fromEnd can be set by the user as usual. The proposed compartmentalized method consists of six phases as described below.

#### A. Initial contig assembly

FPC's BUILD-CONTIGS procedure is run on each clone set. This step generates a "local" physical map for each clone set, composed of contigs and singletons. Clones that are completely contained in other clones are buried by FPC. After the contig assembly, all the local physical maps are concatenated into a single project. When a soft pre-clustering is used, a complication is that FPC cannot handle multiple instances of a clone with the same name. We resolve this problem by adding a distinct suffix so that we can distinguish multiple copies of the same clone. The renaming process is transparent to users and in the final physical map, all clones will have their original (unique) names.

#### B. Redundancy removal

If soft clustering is employed in the pre-clustering step, the process of concatenating local physical maps can result in redundant clones and contigs.

We call a contig *redundant *if all of its clones (excluding Q-clones) are completely contained in another contig. By computing the number of common clones between all contig pairs, redundant contigs are detected. In a group of several identical contigs, only one of them is kept alive. All Q-clones that belong to a redundant contig are moved to the singleton set.

We call a clone *redundant *if either (1) it is a singleton and it also occurs in a contig or (2) it occurs multiple times in the set of singleton clones or (3) it occurs multiple times in the same contig. In a group of several identical clones, only one of them is kept alive.

#### C. FPC processing

In this phase, the main FPC procedures are run iteratively on the merged map as discussed in [27]. Steps (C2)-(C5) are repeated until convergence. For more details on FPC functionalities, see [18,26].

#### (C1) Resolve Q-clones

We run the FPC procedure DQER that reduces the number of Q-clones in an attempt to split the incorrectly merged contigs. DQER runs the CB algorithm on contigs that contain more than *q*% of Q-clones, where *q *is an input parameter.

#### (C2) Merge contigs

We execute the FPC procedure END-MERGER that merges two contigs *A *and *B *if *M *distinct pairs of *end clones*, one of which is in *A *and the other in *B*, match each other with a Sulston score lower than the cutoff value. A clone in a contig is an *end clone *if it is within fromEnd CB units from one of the ends of the contig, where fromEnd is an input parameter [27]. To avoid making wrong merges early in the process, we run END-MERGER with increasingly lower values of *M *(6 for the first iteration, 4 for the second, and 3 for the following iterations).

#### (C3) Eliminate redundant contigs and clones

See Phase B.

#### (C4) Rebuild contigs

We execute the FPC procedure REBUILD-CONTIGS at this point because END-MERGER does not update the CB map (in FPC v8.0 or above, see [27]). REBUILD-CONTIGS executes the CB algorithm on the current version of the contigs in order to improve the clone ordering.

#### (C5) Resolve Q-clones

See Step (C1).

#### D. Post-processing

In this fourth phase, we merge contigs with a novel algorithm described below and then we remove possible redundancies present in the physical map. Steps D2-D4 are repeated a few times until convergence. Phase D is needed only when soft clustering is used in the pre-clustering step.

#### (D1) Eliminate redundant Q-clones

A *redundant Q-clone *is a Q-clone that occurs as a non-Q-clone in another contig. The removal of redundant Q-clones is performed only in this phase, since DQER resolves most of the Q-clones in the FPC processing phase (see Phase C).

#### (D2) Merge contigs

Recall that END-MERGER merges two contigs if a given number of their end clones overlap with a Sulston score lower than the cutoff. However, in the compartmentalized method, contigs may still share several common clones. Contigs that share a large number of clones should be merged, so we designed an algorithm called MERGE-SIMILAR-CONTIGS that works as follow. For all contig pairs (*c*_*j*_, *c*_*k*_) for which *S *= *c*_*j *_∩ *c*_*k *_≠ ∅, the probability that they share clones in *S *(according to an independent and identically distributed (i.i.d.) model) is



where *M *is the multiset of all clones in the physical map, and  is the number of copies of the *i*-th element in *S*. Given these probabilities and a specified threshold *T*_*p*_, we build a directed acyclic graph *G *= (*V, E*), where *V *is the set of contigs that share at least one clone with some other contig, and *E *= {(*u*, *v*)|*p*(*u*, *v*) ≤ *T*_*p *_and |*u*| ≤ |*v*|}. When *p*(*u, v*) ≤ *T*_*p *_and |*u*| = |*v*|, source and destination of the edge are selected according to the lexicographical order of names of *u *and *v*. We merge contig *u *to contig *m*(*u*), where



MERGE-SIMILAR-CONTIGS is run until no further merging is possible. As in step (C2), the threshold *T*_*p *_is increased at each iteration until it reaches a user-supplied maximum (0 for the first iteration, 1e-30 for the second, and 1e-15 for the following iterations).

#### (D3) Eliminate redundant contigs and clones

See Phase B.

#### (D4) Move redundant clones to the singleton set

After merging contigs, there may be still some clones that occur in multiple contigs. Since the location of these clones in the physical map is ambiguous, they are moved to the set of singletons.

#### E. Singleton processing

Up to this phase, singleton clones that belong to distinct clone sets have not being processed. In order to check whether singleton clones are overlapping, an additional round of contig assembly is performed by running FPC's BUILD-CONTIGS procedure on the singletons set.

#### F. Finalizing

In this phase, some final adjustments are carried out on the physical map. Specifically, we reorder the clones (see Step C4) and try to resolve any Q-clone introduced in the last phase (see Step C1).

#### Implementation

The compartmentalized assembler is implemented in C/C++ and Perl. Contig assembly is performed by FPC, which is implemented in C [18]. Our software tool compiles and runs under Linux and Mac OS.

### Dataset

We used the genomic data of two plants, namely barley and rice, to compare our compartmentalized approach to the standard FPC assembly.

For barley, we used OLIGOSPAWN[28] to design 12,467 36-mer oligonucleotide probes from a dataset of 53,799 barley unigenes obtained from HarvEST website [29]. A *unigene *is obtained as a product of assembling several ESTs. Probes were grouped in 70 pools of usually 192 probes each, with a maximum of 310 overgos in a single pool.

**Figure 6:**
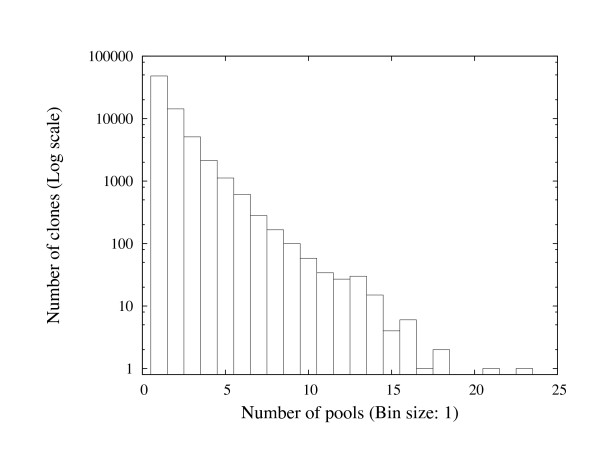
**The histogram of the number of occurrences of barley clones in the probe pools**.

The barley BAC library screened against the pools of overgos is a Morex library covering 6.3 genome equivalents [30]. 83,831 BAC clones were detected as gene-bearing after the hybridization experiment with the probes. Figure 6 shows the histogram of number of occurrences of the barley clones in the probe pools. As shown in the figure, the majority of the barley clones occur in a few pools only.

Restriction fingerprint data of gene-bearing BAC clones of barley were obtained by using High Information Content Fingerprinting (HICF) as part of our NSF funded project (manuscript in preparation). Exactly 61,647 of these clones were successfully fingerprinted (M.C. Luo, personal communication). The average insert size of these clones is 106 kb, and the average number of bands is 92.

Since the barley genome has not been sequenced yet, we had to resort to an organism with a known genome for our comparative evaluations. We used agarose gel-based restriction fingerprint data and the manually edited physical map of rice obtained from Arizona Genomic Institute [31,32] for this purpose. The restriction fingerprint data were real, but the hybridization fingerprint data were obtained by carrying out the hybridization of rice BAC clones to 36-mer rice oligonucleotide probes *in silico*.

**Figure 7:**
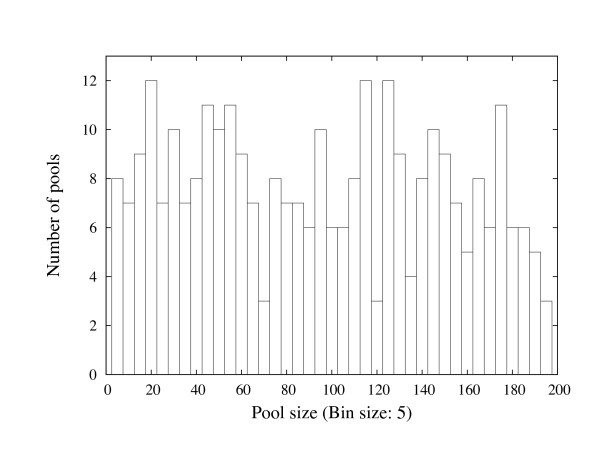
**The histogram of the rice probe pool sizes**.

We used OLIGOSPAWN to design 36-mer unique oligonucleotide probes from rice unigene dataset (build 62) obtained from NCBI [33] containing 46,381 unigenes. For about 70% of unigenes, at least one unique probe was designed. We generated 305 pools of rice oligonucleotide probes by randomly selecting (based on uniform distribution) at most 200 probes in each pool. The histogram of the probe pool sizes is shown in Figure 7.

To model the hybridization, we decided that if a probe had a perfect match to a BAC clone with 30 or more consecutive bases (out of 36), we considered it a positive hybridization. We also introduced noise in the hybridization experiment (i.e., false positive, false negative hybridization). To model the noise, *FN*% of clone-probe hits were discarded to generate false negative, and *FP*% of clone-probe pairs that did not hybridize were considered positive to generate false positive hybridization errors.

**Figure 8:**
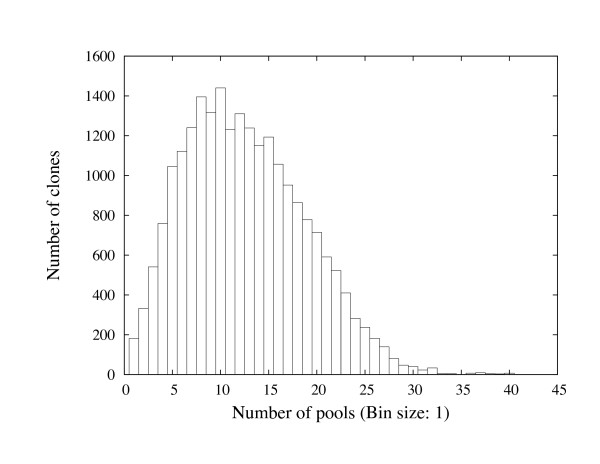
**The histogram of the number of occurrences of rice clones in the probe pools**.

Figure 8 shows the histogram of number of occurrences of the rice clones in the probe pools. Compared to the barley clones, the majority of the rice clones are present in more than ten pools.

In order to carry out the hybridization of rice BAC clones to oligonucleotide probes *in silico*, we obtained the sequences of rice clones indirectly by uniquely locating their BAC end sequences (BESs) obtained from Arizona Genomic Institute [6,34] on the rice genome.

There were 59,430 rice BAC clones for which BAC-end sequences (BESs) were available, but only 65% of them had both BESs sequenced. In order to uniquely locate BAC clones in the rice genome, we BLASTed the BESs against the rice genome (fourth release [6,35-37]) and filtered out low-scoring BLAST hits. We set e-value and word size parameters to 1e-100 and 11, respectively. We also enabled filtering and allowed gaps. If a BAC clone had at least one pair of good BLAST hits, it was selected for further analysis. For each selected BAC clone, we checked all possible pairs of left and right BES hits. The coordinates were assigned only when there was only one pair for which (1) the hits were on the same chromosome, (2) the distance between them was consistent with the typical length of a BAC clone, and (3) the orientations of the alignment for the two ends were opposite to each other. If more than one pair met the criteria (1–3), we declared that the location of that clone in the genome was ambiguous, thus could not be determined. Following this procedure, we obtained 26,469 rice BAC clones for which the sequence could be uniquely determined.

We verified the correctness of this procedure by matching the sequences obtained by our method against the small subset of 3,413 BAC clones sequenced by the International Rice Genome Sequencing Project (IRGSP) [38]. When we aligned the sequences obtained by our method against the actual sequenced BAC clones using MUMmer [39], only 0.8% of the sequences turned out to be misaligned.

The final dataset contained clones for which a unique location in the rice genome was determined and the restriction and hybridization fingerprint data were available. It contained 22,486 clones (about 10× genome equivalence), where the average insert size is 145 kb and the average number of bands is 29.

We applied both the standard and compartmentalized method to the rice and barley data. We generated a compartmentalized map of rice for each pre-clustering method (namely, sHYB, HYB, RESTR, and RAND). Since the purpose of pre-clustering with RESTR and RAND are just to demonstrate the performance of the method when no additional information is available, we generated compartmentalized maps of barley for sHYB and HYB methods only. The compartmentalized maps of rice with HYB and sHYB rely on *in silico *hybridization fingerprint data that contains 5% false positive and false negative hybridizations. On the rice clones, the thresholds for PNC and Sulston score used in HYB and RESTR were 1.45 and 1e-12, respectively. On barley, the threshold for PNC score used in HYB clones was 1.7. No parameter was needed for sHYB and RAND. Statistics of clusters for other choices of parameters are reported in Table 1. The *tolerance *parameter used in the compartmentalized assembler is the same one that was used in the standard method. This is because the tolerance should be set according to the quality of restriction fingerprint data [26] and both methods use the same data. The *cutoff *parameter is also the same in both methods because the cutoff only depends on genome size [22] and genome composition [26]. We set these two parameters according to the specifications of the physical mapping projects of rice [32] and barley (manuscript in preparation).

**Table 1:** Pre-clustering statistics

	Method	Threshold	Clusters	Singletons	Max. cluster size
Rice	sHYB	N/A	305	0	2,533

Rice	HYB	1.1	2	21	22,459
		1.2	50	36	22,078
		1.35	853	286	10,773
		1.4	1,361	528	4,052
		1.45	1,863	880	359
		1.5	2,341	1,405	143

Rice	RESTR	1e-8	128	125	21,663
		1e-10	1,247	357	258
		1e-12	1,929	855	122
		1e-15	2,959	3,007	79
		1e-17	3,421	5,799	67
		1e-20	3,085	12,399	59
		1e-25	680	20,925	12
		1e-30	8	22,470	2

Rice	RAND	N/A	1,901	0	113

Barley	sHYB	N/A	70	0	1,413

Barley	HYB	1.5	311	141	14,471
		1.6	318	211	14,375
		1.7	2,124	988	2,880

### Physical map statistics

Table 2 reports some statistics about the physical maps of rice and barley. The physical maps of barley contain more Q-contigs (i.e., contigs that contain at least one Q-clone) than the physical maps of rice due to the specific fingerprinting method used [27].

**Table 2:** Physical map statistics

	Clones	Contigs	Singl.	Q-contigs
Rice FPC Standard	22,486	1,918	860	8
Rice Comp. sHYB	22,486	2,032	1,156	6
Rice Comp. HYB	22,486	2,070	2,593	3
Rice Comp. RESTR	22,486	1,918	860	8
Rice Comp. RAND	22,486	1,994	862	6

Barley FPC Standard	61,647	8,852	8,821	869
Barley Comp. sHYB	61,647	9,316	10,866	601
Barley Comp. HYB	61,647	9,376	13,024	**489**

According to the statistics in Table 2, the compartmentalized assembler produces physical maps, which contain fewer Q-contigs, but also more contigs and singletons than the maps produced by the standard method. As expected, our compartmentalized assembler is more "stringent" than the standard method because it restricts clones that are in different clusters from being assembled in the same contig. This stringency reduces the number of mis-assembled (i.e., falsely merged) contigs as will be discussed in Evaluation I in Comparative Evaluations of Rice Physical Maps section.

The statistics of the compartmentalized maps of rice obtained by applying RESTR or RAND in the pre-clustering phase and the standard map are almost identical. The maps themselves were also almost identical, which suggests that the compartmentalized assembly with RESTR and RAND perform like the standard method, i.e., pre-clustering with RESTR and RAND brings no benefits. This conclusion was somewhat expected because no additional information is exploited in RESTR and RAND.

We also observe that about 99.6% of the singletons in the standard map of rice are also singletons in the maps using sHYB and HYB pre-clustering. For barley, about 92% and 93% of the singletons in the standard map are also singletons in the maps using sHYB and HYB pre-clustering, respectively. When we analyzed the extra singletons in the rice map obtained with sHYB pre-clustering, we determined that about 81% of these extra singleton clones were *misplaced *in the standard physical map of rice (see Evaluation I in Comparative Evaluations of Rice Physical Maps section for definition of a misplaced clone). In addition, 99% of the misplaced clones in the standard map of rice are singletons in the HYB rice map. The analysis demonstrates that our method is capable of detecting and isolating problematic clones.

### Comparative evaluations of rice physical maps

Since the genomic coordinates of the clones in the rice physical maps are known, more precise comparative evaluations can be carried out for this organism. Specifically, we report on four evaluation metrics to compare the rice physical maps produced by the compartmentalized and standard method, as well as the manually edited map. We were unable to evaluate the quality of the contig assembly in the manually edited physical map of rice (Evaluation I-III), since most of the clones in this map cannot be uniquely located in the rice genome.

#### Evaluation I (Contig assembly)

In this evaluation, we assess the quality of the contig assembly. We considered a contig to be of high quality when most (here 70%) of its clones were truly close to each other in the genome. In order to objectively measure the quality of each contig in the map, we first grouped the clones according to their locations in the genome. Each pair of clones in a given contig were assigned to the same group if they were on the same chromosome and the distance between them was smaller than a predefined threshold. Our tests showed that the choice of specific value for the threshold (in the range 1 kb-100 kb) does not have an impact on the grouping. This suggests that clones are assigned to different groups usually because they reside on different chromosomes (see Table 1 in Additional file 1). In the following evaluation, we show results based on grouping with 1 kb threshold.

After grouping the clones in each contig, we computed the *contig score*, which is defined as the percentage of clones in the largest group. For example, a contig score of 90% means that 90% of the clones in a contig are on the same chromosome and relatively close to each other. Then, an *assembly score *of the whole map was computed as the weighted mean of the contig scores for all contigs in the physical map, using the contig size (i.e., number of clones in a contig) as the weighting factor. The assembly score of each map is shown in Table 3. The results show that the compartmentalized method produces better maps than the standard FPC method when we employ a pre-clustering based on hybridization fingerprints. As discussed earlier, pre-clustering using RESTR or RAND produces results almost identical to the standard map. In this evaluation, we also computed the number of misplaced clones and mis-assembled contigs in each physical map. When the large majority (70% in this evaluation) of the clones in a contig belong to a single group then the other clones in that contig are called *misplaced*. A contig is called *mis-assembled *if it contains at least one misplaced clone. As shown in Table 3, the maps using HYB/sHYB pre-clustering have fewer misplaced clones and mis-assembled contigs than the standard method. We also observe that using the HYB pre-clustering we obtain much fewer misplaced clones and mis-assembled contigs than using the sHYB option.

**Table 3:** The assembly score, the global ordering score, the number of misplaced clones, and the number of mis-assembled contigs for several physical maps of rice

	Assembly score (%)	Misplaced clones	Misass. contigs	Global ordering
FPC Standard	96.43	675	493	0.8252
Comp. sHYB	97.67	343	290	0.8496
Comp. HYB	**99.75**	**10**	**7**	**0.8575**
Comp. RESTR	96.43	675	473	0.8254
Comp. RAND	96.48	668	492	0.8252

A further analysis showed that the set of misplaced clones in the maps using sHYB/HYB pre-clustering is completely contained in the set of misplaced clones in the standard map (see Table 2 in Additional file 1). Moreover, the map using HYB isolates 98.5% of the additional misplaced clones in the standard map to the singleton set. This analysis shows that our method can detect and isolate clones that are otherwise misplaced by the standard method. These misplaced clones are usually responsible for connecting contigs that are not truly overlapping and therefore creating chimeric contigs if left in the assembly.

#### Evaluation II (Clone ordering)

It is well known that due to the noise in the restriction fingerprint data, determining the correct ordering of the clones is a challenging problem [26,40]. Nonetheless, since we have the coordinates of rice clones, we can compute a clone ordering score for each contig. We define the *ordering score *of a contig as the absolute value of the Pearson product-moment correlation coeffcient between the *ranking *of its clones in the genome and their order in the contig.

The *rankings *of clones in the genome are obtained from their coordinates if they belong to the same chromosome. If two clones belong to two different chromosomes then the clone with lower chromosome number has lower ranking than the ranking of the other. For this evaluation, we computed a *global ordering score *for each physical map as the weighted mean of the ordering score of all contigs in the physical map, using the contig size as the weighting factor.

The results in Table 3 show that the compartmentalized method produces contigs whose the clone ordering is more accurate than the standard FPC method. Among the compartmentalized maps, the rice map using HYB has the maximum global ordering score. From our experience, we conjecture that the global ordering score is inversely correlated with the number of mis-assembled contigs.

#### Evaluation III (Minimal tiling path)

As mentioned above, the minimal tiling path (MTP) of a physical map is a critical component of clone-by-clone sequencing projects [41,42]. The quality of an MTP critically depends on the overall quality of its physical map. In this evaluation, first we computed an MTP for the all rice physical maps by using the most recent version of FPC (v8.9 as the time of writing) with default parameters. Then, we compared the number of the MTP clones, the coverage of the MTP clones on the genome, and the percentage of the consecutive MTP clones that truly overlap on the genome.

The results shown in Table 4 illustrate that all maps use approximately the same number of clones, but the genome coverage for the MTP of the rice HYB map is 1% higher than the MTP of the standard FPC map. We also observe that in the physical map obtained by the standard method a higher number of the consecutive MTP clones do not actually overlap on the genome. In other words, the MTP of the standard map contains more gaps than the MTP for the rice HYB and sHYB maps.

**Table 4:** MTP-based evaluation

	MTP clones	Coverage (%)	Overlaps (%)
FPC Standard	2,791	84.89	84.31
Comp. sHYB	2,874	85.66	86.94
Comp. HYB	2,810	**85.89**	**94.05**
Comp. RESTR	2,792	84.85	84.33
Comp. RAND	2,856	85.08	83.75

#### Evaluation IV (Overlap detection)

In our final evaluation, we focused on the set of clones that overlap in the genome. For each pair of clones that are actually overlapping, we checked whether they were in the same contig (counted as true positive, or TP) or not (counted as false negative, or FN). If one or both clones were in the singleton set, this pair was added to the singletons count. Only clone pairs that overlap by at least 100 kb were considered in the evaluation because, FPC can join two clones only if they overlap by at least 70% of their length (70% of the BAC clone size is about 100 kb) [26].

The results in Table 5 show that although the true positive rate in the rice standard map is higher than the compartmentalized map using HYB or sHYB, the former suffers from a much higher false negative rate. We also observe that the manually edited physical map is much better than both compartmentalized and standard physical maps. This is not surprising given that the manually edited physical map of rice is the result of years of curation from experts. In closing, we should keep in mind that this evaluation metric favors physical maps with smaller number of contigs. In the extreme, the pathological physical map in which all clones were assigned to one single contig would beat, according to this evaluation, all the maps considered here.

**Table 5:** Evaluation results for standard, compartmentalized, and manually edited physical maps of rice (based on overlapping clones) and barley (based on genetic markers)

	TP (%)	FN (%)	Singl. (%)
Rice FPC Standard	88.91	8.53	2.56
Rice Comp. sHYB	87.90	7.05	5.05
Rice Comp. HYB	83.87	**1.90**	14.23
Rice Comp. RESTR	88.91	8.53	2.56
Rice Comp. RAND	87.80	9.64	2.56
Rice Manual	**92.09**	7.26	0.65

Barley Standard	82.27	9.63	8.10
Barley Comp. sHYB	**82.55**	9.25	8.20
Barley Comp. HYB	72.55	**9.06**	18.40

### Comparative evaluation of barley physical maps

Since the barley genome has not been sequenced yet, none of the evaluations discussed above can be carried out. We were able, however, to obtain from the Institute of Plant Genetics and Crop Plant Research (IPK) a list of 340 sets of BAC clones that are known to hybridize to a single oligonucleotide probe. The list was extended to 731 sets of BACs by incorporating BAC-gene deconvolution data obtained from the barley genetic map. The assumption is that the clones in each set should overlap each other because they are all positive for a single probe. In practice, this is not necessarily true for all the clones in the sets due to noise in the hybridization experiment, or because BAC clones overlap a repeat region or a gene family. Although this evaluation is not 100% reliable, it was the best validation of the barley map available.

The evaluation was carried out as follows. For each clone set identified by a probe, we first computed the contig ID that contains the majority of the clones in the set. Then for all clones in the set, we computed the number of clones that were either in that contig (counted as TP), or in another contig (counted as FN), or in the singleton set.

The results shown in Table 5 illustrate that the barley HYB has the fewest errors among all barley maps. In other words, the compartmentalized method with HYB is able to isolate some clones to the singleton set that would have otherwise been misplaced by the standard method.

We also computed the number of *gaps *in the physical maps of barley. If a set of clones that should belong to one contig is distributed in more than one contig, we record this event as a *gap*. More precisely, for each list of clones that are assumed to be overlapping, we computed the number of contigs that they belong to and computed the number of gaps for each list. According to the results, barley HYB has 240 gaps, barley sHYB has 293 gaps, and the standard map has 280 gaps. The results show that number of gaps correlates with the FN rate of the maps.

## Conclusion

We proposed a novel compartmentalized approach to the construction of physical maps from fingerprinted clones. The compartmentalized method exploits both the restriction and hybridization fingerprint data, which allows it to construct more accurate physical maps. Consequently, we argue that the compartmentalized method reduces the amount of manual editing that is an inevitable step in any physical mapping project. Additionally, we showed that the MTP produced from the compartmentalized physical map is more reliable, and that should help clone-by-clone sequencing projects and *de novo *sequence assembly with short reads. The software is available in the public domain at .

## Authors' contributions

SB developed the method, performed the *in silico *hybridization, generated the experimental results, and wrote the manuscript. TJC was involved in developing the method and writing the manuscript. SL was involved in developing the method, generating the experimental results, and writing the manuscript.

## Supplementary Material

Additional file 1 **Supplementary Tables.doc**. Contains two supplementary tables. The first table shows the percentage of cases that two clones which are in same contig, but assigned to different clusters are on the same chromosome or not. The second table lists misplaced clones in Rice HYB, Rice sHYB, and Rice FPC Standard maps. Click here for file
