# Supplementary material for: A compartmentalized approach to the assembly of physical maps
Source: BMC Bioinformatics. 2009 Jul 15;10:217. doi: 10.1186/1471-2105-10-217 (PMC2717093; doi:10.1186/1471-2105-10-217)
Supplement: Additional file 1 — Supplementary Tables.doc. Contains two supplementary tables. The first table shows the percentage of cases that two clones which are in same contig, but assigned to different clusters are on the same chromosome or not. The second table lists misplaced clones in Rice HYB, Rice sHYB, and Rice FPC Standard maps. [file 1471-2105-10-217-S1.doc]

| Threshold | Physical map | Same chr. (%) | Different chr. (%) |
| --- | --- | --- | --- |
| 1 kb | Rice HYB | 0 | 100 |
| 100 kb | Rice HYB | 0 | 100 |
| 1 kb | Rice sHYB | 9.8 | 91.2 |
| 100 kb | Rice sHYB | 9.8 | 91.2 |
| 1 kb | Rice REST | 11.1 | 88.9 |
| 100 kb | Rice REST | 11.1 | 88.9 |
| 1 kb | Rice RAND | 10.9 | 89.1 |
| 100 kb | Rice RAND | 10.9 | 89.1 |

Supplementary Table 1 Percentage of cases in which two clones that are in same contig are assigned to different clusters. Case 1: clones are on same chromosome but apart from each other (Same chr. column), Case 2: clones are on different chromosomes (Different chr.) As results show the choice of threshold has no impact on the clustering and usually clones are assigned to different clusters because they reside on different chromosomes.

| Clones | Rice HYB | Rice sHYB | Rice FPC Standard |
| --- | --- | --- | --- |
| a0007M05 | + | + | + |
| a0037I17 | + | + | + |
| a0037M05 | + | + | + |
| a0047D01 | + | + | + |
| a0047L09 | + | + | + |
| a0060J13 | + | + | + |
| a0002J13 | + | - | + |
| a0023F13 | + | - | + |
| a0037K17 | + | - | + |
| a0058F08 | + | - | + |
| a0001F21 | - | + | + |
| a0002B05 | - | + | + |
| a0002B12 | - | + | + |
| a0002B13 | - | + | + |
| a0002B23 | - | + | + |
| a0002D05 | - | + | + |
| a0002D07 | - | + | + |
| a0002D09 | - | + | + |
| a0002D16 | - | + | + |
| a0002D23 | - | + | + |
| a0002F01 | - | + | + |
| a0002F03 | - | + | + |
| a0002F19 | - | + | + |
| a0002F21 | - | + | + |
| a0002H01 | - | + | + |
| a0002H07 | - | + | + |
| a0002H09 | - | + | + |
| a0002H15 | - | + | + |
| a0002H19 | - | + | + |
| a0002J05 | - | + | + |
| a0002L09 | - | + | + |
| a0002L23 | - | + | + |
| a0002N07 | - | + | + |
| a0002N09 | - | + | + |
| a0002N13 | - | + | + |
| a0002N17 | - | + | + |
| a0002N19 | - | + | + |
| a0002O09 | - | + | + |
| a0002P07 | - | + | + |
| a0002P17 | - | + | + |
| a0002P21 | - | + | + |
| a0007M01 | - | + | + |
| a0008J04 | - | + | + |
| a0008J10 | - | + | + |
| a0008J14 | - | + | + |
| a0008J18 | - | + | + |
| a0008J24 | - | + | + |
| a0008L10 | - | + | + |
| a0008L14 | - | + | + |
| a0008L22 | - | + | + |
| a0008L24 | - | + | + |
| a0008N10 | - | + | + |
| a0008N18 | - | + | + |
| a0008P10 | - | + | + |
| a0008P14 | - | + | + |
| a0008P16 | - | + | + |
| a0008P22 | - | + | + |
| a0009M09 | - | + | + |
| a0010C22 | - | + | + |
| a0010J08 | - | + | + |
| a0010J10 | - | + | + |
| a0010L07 | - | + | + |
| a0010N06 | - | + | + |
| a0010N20 | - | + | + |
| a0010N22 | - | + | + |
| a0010P02 | - | + | + |
| a0010P06 | - | + | + |
| a0010P22 | - | + | + |
| a0011I03 | - | + | + |
| a0011I05 | - | + | + |
| a0011I07 | - | + | + |
| a0011I15 | - | + | + |
| a0011I17 | - | + | + |
| a0011K13 | - | + | + |
| a0011M09 | - | + | + |
| a0011M23 | - | + | + |
| a0011O09 | - | + | + |
| a0011O13 | - | + | + |
| a0011O15 | - | + | + |
| a0011O17 | - | + | + |
| a0012A19 | - | + | + |
| a0012A20 | - | + | + |
| a0012M09 | - | + | + |
| a0016H01 | - | + | + |
| a0016H11 | - | + | + |
| a0019N07 | - | + | + |
| a0019N13 | - | + | + |
| a0019N19 | - | + | + |
| a0019P21 | - | + | + |
| a0019P23 | - | + | + |
| a0020H11 | - | + | + |
| a0021F23 | - | + | + |
| a0022E02 | - | + | + |
| a0022E06 | - | + | + |
| a0022E10 | - | + | + |
| a0022E14 | - | + | + |
| a0022E16 | - | + | + |
| a0022E18 | - | + | + |
| a0022E20 | - | + | + |
| a0022G02 | - | + | + |
| a0022G10 | - | + | + |
| a0022G20 | - | + | + |
| a0022G24 | - | + | + |
| a0023B01 | - | + | + |
| a0023B13 | - | + | + |
| a0023B14 | - | + | + |
| a0023D08 | - | + | + |
| a0023D11 | - | + | + |
| a0023D13 | - | + | + |
| a0023D16 | - | + | + |
| a0023D21 | - | + | + |
| a0023D22 | - | + | + |
| a0023D24 | - | + | + |
| a0023F11 | - | + | + |
| a0023F22 | - | + | + |
| a0023H02 | - | + | + |
| a0023H04 | - | + | + |
| a0023H08 | - | + | + |
| a0023H10 | - | + | + |
| a0023H12 | - | + | + |
| a0023H17 | - | + | + |
| a0023H21 | - | + | + |
| a0023J16 | - | + | + |
| a0023J18 | - | + | + |
| a0023L04 | - | + | + |
| a0023L06 | - | + | + |
| a0023L10 | - | + | + |
| a0023N20 | - | + | + |
| a0023P11 | - | + | + |
| a0023P12 | - | + | + |
| a0023P17 | - | + | + |
| a0023P18 | - | + | + |
| a0025B22 | - | + | + |
| a0026A07 | - | + | + |
| a0027A22 | - | + | + |
| a0027C06 | - | + | + |
| a0027C08 | - | + | + |
| a0027C22 | - | + | + |
| a0027E06 | - | + | + |
| a0027E14 | - | + | + |
| a0027E24 | - | + | + |
| a0027G02 | - | + | + |
| a0027G08 | - | + | + |
| a0027G10 | - | + | + |
| a0027G22 | - | + | + |
| a0027G24 | - | + | + |
| a0027K16 | - | + | + |
| a0028L02 | - | + | + |
| a0028L14 | - | + | + |
| a0028L18 | - | + | + |
| a0028L22 | - | + | + |
| a0028N02 | - | + | + |
| a0028N08 | - | + | + |
| a0028N10 | - | + | + |
| a0028N12 | - | + | + |
| a0028N14 | - | + | + |
| a0028N16 | - | + | + |
| a0028N22 | - | + | + |
| a0029E17 | - | + | + |
| a0029E18 | - | + | + |
| a0029E20 | - | + | + |
| a0030P09 | - | + | + |
| a0030P11 | - | + | + |
| a0032A18 | - | + | + |
| a0032A20 | - | + | + |
| a0032O22 | - | + | + |
| a0034A05 | - | + | + |
| a0034A07 | - | + | + |
| a0034A09 | - | + | + |
| a0034A11 | - | + | + |
| a0034A17 | - | + | + |
| a0034A21 | - | + | + |
| a0034C19 | - | + | + |
| a0034E03 | - | + | + |
| a0034E09 | - | + | + |
| a0034E15 | - | + | + |
| a0034E17 | - | + | + |
| a0034E19 | - | + | + |
| a0034G01 | - | + | + |
| a0034G05 | - | + | + |
| a0034G07 | - | + | + |
| a0034G09 | - | + | + |
| a0034G15 | - | + | + |
| a0034G21 | - | + | + |
| a0034I03 | - | + | + |
| a0034I07 | - | + | + |
| a0034I15 | - | + | + |
| a0034K01 | - | + | + |
| a0034K07 | - | + | + |
| a0034K09 | - | + | + |
| a0034M05 | - | + | + |
| a0034M13 | - | + | + |
| a0034O01 | - | + | + |
| a0034O11 | - | + | + |
| a0036J17 | - | + | + |
| a0036P17 | - | + | + |
| a0037A07 | - | + | + |
| a0037A09 | - | + | + |
| a0037A10 | - | + | + |
| a0037A14 | - | + | + |
| a0037A17 | - | + | + |
| a0037A19 | - | + | + |
| a0037A20 | - | + | + |
| a0037B20 | - | + | + |
| a0037C05 | - | + | + |
| a0037C09 | - | + | + |
| a0037C10 | - | + | + |
| a0037C11 | - | + | + |
| a0037C14 | - | + | + |
| a0037C19 | - | + | + |
| a0037E01 | - | + | + |
| a0037E04 | - | + | + |
| a0037E14 | - | + | + |
| a0037G06 | - | + | + |
| a0037G16 | - | + | + |
| a0037G18 | - | + | + |
| a0037G19 | - | + | + |
| a0037G20 | - | + | + |
| a0037I03 | - | + | + |
| a0037I09 | - | + | + |
| a0037I21 | - | + | + |
| a0037I24 | - | + | + |
| a0037K01 | - | + | + |
| a0037K06 | - | + | + |
| a0037M06 | - | + | + |
| a0037M17 | - | + | + |
| a0037O07 | - | + | + |
| a0037O08 | - | + | + |
| a0037O09 | - | + | + |
| a0037O10 | - | + | + |
| a0037O17 | - | + | + |
| a0037O18 | - | + | + |
| a0037O20 | - | + | + |
| a0041O06 | - | + | + |
| a0041O08 | - | + | + |
| a0041O16 | - | + | + |
| a0042P18 | - | + | + |
| a0044A09 | - | + | + |
| a0044C09 | - | + | + |
| a0044E19 | - | + | + |
| a0044I19 | - | + | + |
| a0044K19 | - | + | + |
| a0044O04 | - | + | + |
| a0044O12 | - | + | + |
| a0044O14 | - | + | + |
| a0044O17 | - | + | + |
| a0044O19 | - | + | + |
| a0044O20 | - | + | + |
| a0044O22 | - | + | + |
| a0044O24 | - | + | + |
| a0045E09 | - | + | + |
| a0046K22 | - | + | + |
| a0047B11 | - | + | + |
| a0047B24 | - | + | + |
| a0047D14 | - | + | + |
| a0047D21 | - | + | + |
| a0047D22 | - | + | + |
| a0047F22 | - | + | + |
| a0047H04 | - | + | + |
| a0047H07 | - | + | + |
| a0047H08 | - | + | + |
| a0047H09 | - | + | + |
| a0047H10 | - | + | + |
| a0047H12 | - | + | + |
| a0047J06 | - | + | + |
| a0047J10 | - | + | + |
| a0047J11 | - | + | + |
| a0047J12 | - | + | + |
| a0047J17 | - | + | + |
| a0047J22 | - | + | + |
| a0047L05 | - | + | + |
| a0047N05 | - | + | + |
| a0047N12 | - | + | + |
| a0047N19 | - | + | + |
| a0047P11 | - | + | + |
| a0047P13 | - | + | + |
| a0047P20 | - | + | + |
| a0048B22 | - | + | + |
| a0048D21 | - | + | + |
| a0048H15 | - | + | + |
| a0048H17 | - | + | + |
| a0048H21 | - | + | + |
| a0049E11 | - | + | + |
| a0050B09 | - | + | + |
| a0050G06 | - | + | + |
| a0052D20 | - | + | + |
| a0052F08 | - | + | + |
| a0052H13 | - | + | + |
| a0053C05 | - | + | + |
| a0053I03 | - | + | + |
| a0055G19 | - | + | + |
| a0055H17 | - | + | + |
| a0055I22 | - | + | + |
| a0055P01 | - | + | + |
| a0056C03 | - | + | + |
| a0057P12 | - | + | + |
| a0058A20 | - | + | + |
| a0058I05 | - | + | + |
| a0058I16 | - | + | + |
| a0058J14 | - | + | + |
| a0058K06 | - | + | + |
| a0058L10 | - | + | + |
| a0059L04 | - | + | + |
| a0060D15 | - | + | + |
| a0060F13 | - | + | + |
| a0060F14 | - | + | + |
| a0060H11 | - | + | + |
| a0060J09 | - | + | + |
| a0060J17 | - | + | + |
| a0060L03 | - | + | + |
| a0060L09 | - | + | + |
| a0060L11 | - | + | + |
| b0001F03 | - | + | + |
| b0001F19 | - | + | + |
| b0001H07 | - | + | + |
| b0027E21 | - | + | + |
| b0039P19 | - | + | + |
| b0044G19 | - | + | + |
| b0055E01 | - | + | + |
| b0055E09 | - | + | + |
| b0055E11 | - | + | + |
| b0055E15 | - | + | + |
| b0055G11 | - | + | + |
| b0086B22 | - | + | + |
| b0098A22 | - | + | + |
| b0098C02 | - | + | + |
| b0098C06 | - | + | + |
| b0108N13 | - | + | + |
| b0122B06 | - | + | + |
| b0122B08 | - | + | + |
| b0122B10 | - | + | + |
| b0122B18 | - | + | + |
| b0122D24 | - | + | + |
| b0122F02 | - | + | + |
| b0122H02 | - | + | + |
| b0122H04 | - | + | + |
| b0122H16 | - | + | + |
| b0122H20 | - | + | + |
| b0122J02 | - | + | + |
| b0122J10 | - | + | + |
| b0122J24 | - | + | + |
| b0122L12 | - | + | + |
| b0122P14 | - | + | + |
| b0122P20 | - | + | + |
| a0002B11 | - | - | + |
| a0002B15 | - | - | + |
| a0002B17 | - | - | + |
| a0002B19 | - | - | + |
| a0002B21 | - | - | + |
| a0002D15 | - | - | + |
| a0002D17 | - | - | + |
| a0002D21 | - | - | + |
| a0002F07 | - | - | + |
| a0002H13 | - | - | + |
| a0002H23 | - | - | + |
| a0002J15 | - | - | + |
| a0002J17 | - | - | + |
| a0002J19 | - | - | + |
| a0002L03 | - | - | + |
| a0002L13 | - | - | + |
| a0002N05 | - | - | + |
| a0002O04 | - | - | + |
| a0002O08 | - | - | + |
| a0002O17 | - | - | + |
| a0002P05 | - | - | + |
| a0002P11 | - | - | + |
| a0002P13 | - | - | + |
| a0003K22 | - | - | + |
| a0004H20 | - | - | + |
| a0007M17 | - | - | + |
| a0008J02 | - | - | + |
| a0008J06 | - | - | + |
| a0008J08 | - | - | + |
| a0008J22 | - | - | + |
| a0008L08 | - | - | + |
| a0008L16 | - | - | + |
| a0008N02 | - | - | + |
| a0008N06 | - | - | + |
| a0008N14 | - | - | + |
| a0008N16 | - | - | + |
| a0008N24 | - | - | + |
| a0008P06 | - | - | + |
| a0008P08 | - | - | + |
| a0010N14 | - | - | + |
| a0010N24 | - | - | + |
| a0010P12 | - | - | + |
| a0010P16 | - | - | + |
| a0010P20 | - | - | + |
| a0011I13 | - | - | + |
| a0011I23 | - | - | + |
| a0011K23 | - | - | + |
| a0011M11 | - | - | + |
| a0011O05 | - | - | + |
| a0013J17 | - | - | + |
| a0016H03 | - | - | + |
| a0016H05 | - | - | + |
| a0016H07 | - | - | + |
| a0016H09 | - | - | + |
| a0018E13 | - | - | + |
| a0019N05 | - | - | + |
| a0019N11 | - | - | + |
| a0019N15 | - | - | + |
| a0019N23 | - | - | + |
| a0019P11 | - | - | + |
| a0019P15 | - | - | + |
| a0020D09 | - | - | + |
| a0020D16 | - | - | + |
| a0022E04 | - | - | + |
| a0022E22 | - | - | + |
| a0022E24 | - | - | + |
| a0022G04 | - | - | + |
| a0022G08 | - | - | + |
| a0022G14 | - | - | + |
| a0023B07 | - | - | + |
| a0023B11 | - | - | + |
| a0023B19 | - | - | + |
| a0023B23 | - | - | + |
| a0023D04 | - | - | + |
| a0023D09 | - | - | + |
| a0023D10 | - | - | + |
| a0023D15 | - | - | + |
| a0023D17 | - | - | + |
| a0023D18 | - | - | + |
| a0023F01 | - | - | + |
| a0023F04 | - | - | + |
| a0023F09 | - | - | + |
| a0023F21 | - | - | + |
| a0023H15 | - | - | + |
| a0023J12 | - | - | + |
| a0023L11 | - | - | + |
| a0023L12 | - | - | + |
| a0023L15 | - | - | + |
| a0023L16 | - | - | + |
| a0023L21 | - | - | + |
| a0023N10 | - | - | + |
| a0023N12 | - | - | + |
| a0023N13 | - | - | + |
| a0023N22 | - | - | + |
| a0023P06 | - | - | + |
| a0023P08 | - | - | + |
| a0023P10 | - | - | + |
| a0023P14 | - | - | + |
| a0023P16 | - | - | + |
| a0023P21 | - | - | + |
| a0026L09 | - | - | + |
| a0027A02 | - | - | + |
| a0027A06 | - | - | + |
| a0027A10 | - | - | + |
| a0027A16 | - | - | + |
| a0027A18 | - | - | + |
| a0027A24 | - | - | + |
| a0027C02 | - | - | + |
| a0027C12 | - | - | + |
| a0027C16 | - | - | + |
| a0027C24 | - | - | + |
| a0027E02 | - | - | + |
| a0027E10 | - | - | + |
| a0027G04 | - | - | + |
| a0027G06 | - | - | + |
| a0027G12 | - | - | + |
| a0027I04 | - | - | + |
| a0027I08 | - | - | + |
| a0027I10 | - | - | + |
| a0027I14 | - | - | + |
| a0027K10 | - | - | + |
| a0027K24 | - | - | + |
| a0028L04 | - | - | + |
| a0028L08 | - | - | + |
| a0028L12 | - | - | + |
| a0028L16 | - | - | + |
| a0028L24 | - | - | + |
| a0028N04 | - | - | + |
| a0028N20 | - | - | + |
| a0028N24 | - | - | + |
| a0029E14 | - | - | + |
| a0029E19 | - | - | + |
| a0029E24 | - | - | + |
| a0029J20 | - | - | + |
| a0030P07 | - | - | + |
| a0031J06 | - | - | + |
| a0031J08 | - | - | + |
| a0031P14 | - | - | + |
| a0034A15 | - | - | + |
| a0034A19 | - | - | + |
| a0034E05 | - | - | + |
| a0034G17 | - | - | + |
| a0034I05 | - | - | + |
| a0034I09 | - | - | + |
| a0034I17 | - | - | + |
| a0034K05 | - | - | + |
| a0034K17 | - | - | + |
| a0034K19 | - | - | + |
| a0034M01 | - | - | + |
| a0034M07 | - | - | + |
| a0034M09 | - | - | + |
| a0034M15 | - | - | + |
| a0034M17 | - | - | + |
| a0034M19 | - | - | + |
| a0034M23 | - | - | + |
| a0034O03 | - | - | + |
| a0034O15 | - | - | + |
| a0036A20 | - | - | + |
| a0036M13 | - | - | + |
| a0036N11 | - | - | + |
| a0036N13 | - | - | + |
| a0036P13 | - | - | + |
| a0037A02 | - | - | + |
| a0037A05 | - | - | + |
| a0037A06 | - | - | + |
| a0037A15 | - | - | + |
| a0037A18 | - | - | + |
| a0037A22 | - | - | + |
| a0037B14 | - | + | + |
| a0037C03 | - | - | + |
| a0037C15 | - | - | + |
| a0037E08 | - | - | + |
| a0037E10 | - | - | + |
| a0037E13 | - | - | + |
| a0037E16 | - | - | + |
| a0037E17 | - | - | + |
| a0037E18 | - | - | + |
| a0037E21 | - | - | + |
| a0037E22 | - | - | + |
| a0037E23 | - | - | + |
| a0037G08 | - | - | + |
| a0037G10 | - | - | + |
| a0037G21 | - | - | + |
| a0037I05 | - | - | + |
| a0037I14 | - | + | + |
| a0037I20 | - | - | + |
| a0037K08 | - | - | + |
| a0037K14 | - | - | + |
| a0037K19 | - | - | + |
| a0037K20 | - | - | + |
| a0037K21 | - | - | + |
| a0037M02 | - | - | + |
| a0037M09 | - | - | + |
| a0037M12 | - | - | + |
| a0037M15 | - | + | + |
| a0037M16 | - | - | + |
| a0037M18 | - | - | + |
| a0037M24 | - | - | + |
| a0037O05 | - | - | + |
| a0037O06 | - | - | + |
| a0037O12 | - | - | + |
| a0037O21 | - | - | + |
| a0037O22 | - | - | + |
| a0041O10 | - | - | + |
| a0042E17 | - | - | + |
| a0044A03 | - | - | + |
| a0044A05 | - | - | + |
| a0044A13 | - | - | + |
| a0044A15 | - | - | + |
| a0044A19 | - | - | + |
| a0044C05 | - | - | + |
| a0044C11 | - | - | + |
| a0044C19 | - | - | + |
| a0044C21 | - | - | + |
| a0044C23 | - | - | + |
| a0044E15 | - | - | + |
| a0044E17 | - | - | + |
| a0044E23 | - | - | + |
| a0044I17 | - | - | + |
| a0044K17 | - | - | + |
| a0044K23 | - | - | + |
| a0044O02 | - | - | + |
| a0044O10 | - | - | + |
| a0044O11 | - | - | + |
| a0044O15 | - | - | + |
| a0044O23 | - | - | + |
| a0045C09 | - | - | + |
| a0045E07 | - | - | + |
| a0045K17 | - | - | + |
| a0046K20 | - | - | + |
| a0047B01 | - | - | + |
| a0047B05 | - | - | + |
| a0047B06 | - | - | + |
| a0047B10 | - | - | + |
| a0047B14 | - | - | + |
| a0047D04 | - | - | + |
| a0047D06 | - | - | + |
| a0047D11 | - | - | + |
| a0047D12 | - | - | + |
| a0047D13 | - | - | + |
| a0047D15 | - | - | + |
| a0047D18 | - | - | + |
| a0047D23 | - | - | + |
| a0047F20 | - | - | + |
| a0047H11 | - | - | + |
| a0047H20 | - | - | + |
| a0047H21 | - | - | + |
| a0047H22 | - | - | + |
| a0047H24 | - | - | + |
| a0047J19 | - | - | + |
| a0047J20 | - | - | + |
| a0047L07 | - | - | + |
| a0047L11 | - | - | + |
| a0047L17 | - | - | + |
| a0047L24 | - | - | + |
| a0047N10 | - | - | + |
| a0047N17 | - | - | + |
| a0047N21 | - | - | + |
| a0047P01 | - | - | + |
| a0047P05 | - | - | + |
| a0047P06 | - | - | + |
| a0047P16 | - | - | + |
| a0047P19 | - | - | + |
| a0047P22 | - | - | + |
| a0047P23 | - | - | + |
| a0048D17 | - | - | + |
| a0048D19 | - | - | + |
| a0048D23 | - | - | + |
| a0048F07 | - | - | + |
| a0048H19 | - | - | + |
| a0048L13 | - | - | + |
| a0048L19 | - | - | + |
| a0048L21 | - | - | + |
| a0048L23 | - | - | + |
| a0050F21 | - | - | + |
| a0051K22 | - | - | + |
| a0052G12 | - | - | + |
| a0052L03 | - | - | + |
| a0052P10 | - | - | + |
| a0053C04 | - | - | + |
| a0053C07 | - | - | + |
| a0053G23 | - | - | + |
| a0053K01 | - | - | + |
| a0053K12 | - | - | + |
| a0055D19 | - | - | + |
| a0055F08 | - | - | + |
| a0056E24 | - | - | + |
| a0056G02 | - | - | + |
| a0056L05 | - | - | + |
| a0056N16 | - | - | + |
| a0057E09 | - | - | + |
| a0057G19 | - | - | + |
| a0057P10 | - | - | + |
| a0058C12 | - | - | + |
| a0058D04 | - | - | + |
| a0058G01 | - | - | + |
| a0058I21 | - | - | + |
| a0059E10 | - | - | + |
| a0059P12 | - | - | + |
| a0059P20 | - | - | + |
| a0060D11 | - | - | + |
| a0060F15 | - | - | + |
| a0060H15 | - | - | + |
| b0019E18 | - | - | + |
| b0032D21 | - | - | + |
| b0032F09 | - | - | + |
| b0039P15 | - | - | + |
| b0055C11 | - | - | + |
| b0055C21 | - | - | + |
| b0055E21 | - | - | + |
| b0055G07 | - | - | + |
| b0055G09 | - | - | + |
| b0122B12 | - | - | + |
| b0122B14 | - | - | + |
| b0122B16 | - | - | + |
| b0122B20 | - | - | + |
| b0122D08 | - | - | + |
| b0122D10 | - | - | + |
| b0122D20 | - | - | + |
| b0122F12 | - | - | + |
| b0122F22 | - | - | + |
| b0122H22 | - | - | + |
| b0122J12 | - | - | + |
| b0122J14 | - | - | + |
| b0122L16 | - | - | + |
| b0122L18 | - | - | + |
| b0122N06 | - | - | + |
| b0122N20 | - | - | + |
| b0122P06 | - | - | + |
| b0122P10 | - | - | + |
| b0122P18 | - | - | + |

Supplementary Table 2 List of misplaced clones in Rice HYB, Rice sHYB, and Rice FPC Standard Maps. ‘+’ means that the clone is misplaced in the specified map, ‘-’ means that the clone is not misplaced in the specified map. The table shows that all misplaced clones in HYB/sHYB maps are completely contained in the set of misplaced clones in the standard map.
